# Fueling Gut Microbes: A Review of the Interaction between Diet, Exercise, and the Gut Microbiota in Athletes

**DOI:** 10.1093/advances/nmab077

**Published:** 2021-07-06

**Authors:** Riley L Hughes, Hannah D Holscher

**Affiliations:** Department of Food Science and Human Nutrition, University of Illinois at Urbana-Champaign, Urbana, IL, USA; Department of Food Science and Human Nutrition, University of Illinois at Urbana-Champaign, Urbana, IL, USA; Division of Nutrition Sciences, University of Illinois at Urbana-Champaign, Urbana, IL, USA

**Keywords:** microbiome, athletic performance, gastrointestinal health, protein, carbohydrates, prebiotics, probiotics

## Abstract

The athlete's goal is to optimize their performance. Towards this end, nutrition has been used to improve the health of athletes’ brains, bones, muscles, and cardiovascular system. However, recent research suggests that the gut and its resident microbiota may also play a role in athlete health and performance. Therefore, athletes should consider dietary strategies in the context of their potential effects on the gut microbiota, including the impact of sports-centric dietary strategies (e.g., protein supplements, carbohydrate loading) on the gut microbiota as well as the effects of gut-centric dietary strategies (e.g., probiotics, prebiotics) on performance. This review provides an overview of the interaction between diet, exercise, and the gut microbiota, focusing on dietary strategies that may impact both the gut microbiota and athletic performance. Current evidence suggests that the gut microbiota could, in theory, contribute to the effects of dietary intake on athletic performance by influencing microbial metabolite production, gastrointestinal physiology, and immune modulation. Common dietary strategies such as high protein and simple carbohydrate intake, low fiber intake, and food avoidance may adversely impact the gut microbiota and, in turn, performance. Conversely, intake of adequate dietary fiber, a variety of protein sources, and emphasis on unsaturated fats, especially omega-3 (ɷ-3) fatty acids, in addition to consumption of prebiotics, probiotics, and synbiotics, have shown promising results in optimizing athlete health and performance. Ultimately, while this is an emerging and promising area of research, more studies are needed that incorporate, control, and manipulate all 3 of these elements (i.e., diet, exercise, and gut microbiome) to provide recommendations for athletes on how to “fuel their microbes.”

## Teaser

**Statement of Significance**: This review provides a comprehensive evaluation of the current evidence for the effects of diet, as it pertains to athletic performance, on the gut microbiota, and the potential for the gut microbiota to impact athletic performance as a result of diet-induced modifications.

## Introduction

The human body integrates thousands of biochemical processes to manifest the various aspects of its metabolic phenotype. The athlete's goal is to optimize this complex system to enhance performance. Nutrition has long been used as a tool by athletes to feed their brains, bones, muscles, and cardiovascular system to foster peak performance (1). However, recent scientific advances suggest that nutrition may also influence athletic performance via the gut and the trillions of microorganisms that inhabit this ecosystem (**Text Box 1**) (2–4). Importantly, diet affects the microbial community within the gut (5–7). As a result, the gut microbiota mediates and modulates many of the effects of diet and nutrition and health, such as the risk of chronic diseases including obesity, type 2 diabetes, and cardiovascular disease (8, 9). However, athletes are interested not only in preventing disease but also in optimizing health and performance.

Text Box 1. Definitions

Gut microbiota: the collection of microorganisms, including bacteria, archaea, fungi, and viruses, in the gut.

Gut microbiome: the collection of genetic information contained within the microbiota that provides information about what microorganisms are present as well as the functional capacity of the ecosystem.

Physical activity: any body movement produced by skeletal muscles that results in energy expenditure.

Exercise: a subcategory of physical activity that encompasses planned, structure, and repetitive movement and has as a final or an intermediate objective the improvement in or maintenance of physical fitness.

References: 3, 4.

**FIGURE 1:**
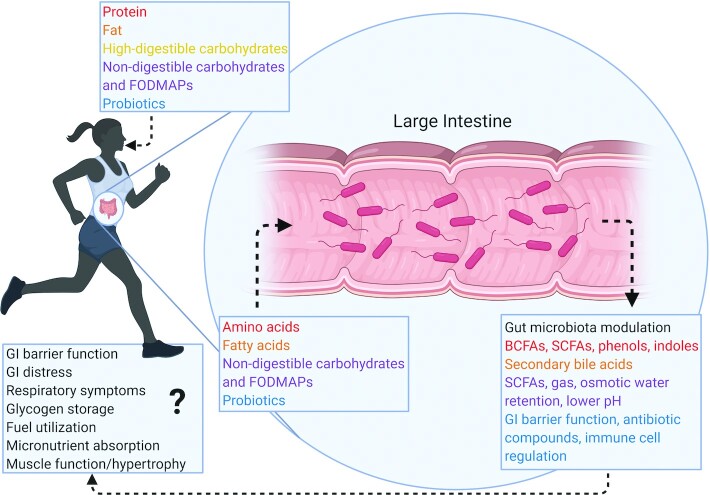
Fueling your microbes for athletic performance. Sport-centric and gut-centric dietary strategies both modulate the composition and function of the gut microbiota, which may then mediate or modulate the effects of these dietary strategies on athletic performance. Human digestive processes produce amino acids and fatty acids from ingested protein and fat, respectively, while nondigestible carbohydrates make it down to the large intestine intact. These components as well as ingested supplements such as probiotics then interact with the gut microbiota, which produces metabolites that influence local gastrointestinal barrier function as well as systemic functions such as glycogen storage, fuel utilization, and muscle function that have the potential to affect athletic performance. BCFA, branched-chain fatty acid; GI, gastrointestinal; FODMAP, fermentable oligo-, di-, monosaccharides and polyols.

Given the gut microbiota's potential to influence athletic performance and its responsiveness to diet, “fueling your microbes” should be seen as a strategy for athletes attempting to optimize performance. Therefore, this report aims to provide a comprehensive review of research on *1*) common dietary strategies utilized by athletes and their effects on the gut microbiota and *2*) dietary strategies utilized to improve gastrointestinal health and effects on athletic performance (**Figure 1**). This review summarizes clinical research investigating connections between the gut microbiota/microbiome and exercise since 2008. However, research on the gut microbiota/microbiome or exercise from any time was included when necessary to provide context, including mechanisms of action. This review aims to synthesize nutrition, exercise, and gut microbiota research to highlight what is known, gaps in the literature, and future directions for research to optimize the interaction between diet, sports, and the gut microbiota for health and athletic performance.

## The Effects of Diet and Sport on the Gut Microbiome

Diet affects the gut microbiota composition and fluctuations (7, 10) over both short (5) and long (6) time frames. In addition to well-studied nutrients like fiber (11), the effects of specific foods (e.g., nuts, avocados) (12, 13) and dietary patterns (e.g., Mediterranean diet pattern) (14) are also recognized. Recent reviews have detailed how exercise influences the gut microbiota, depending on the type, intensity, and exercise duration (2, 10, 15–17). Indeed, evidence suggests that exercise increases α-diversity and microbial metabolites such as SCFAs (2). Effects of exercise on individual taxa are variable but typically reveal increases in commensal taxa such as *Bifidobacterium, Lactobacilli*, and *Akkermansia* (2). The gut microbiota may also influence exercise performance by producing metabolites such as SCFAs, which are utilized as fuel by colonocytes or absorbed into systemic circulation (acetate, 36%; propionate, 9%; butyrate 2%) (18). In skeletal muscle, SCFAs can be oxidized, incorporated into glucose via gluconeogenesis, or increase the bioavailability of glucose, glycogen, and fatty acids during exercise (2, 18–23). SCFAs also contribute to increased blood flow, insulin sensitivity, skeletal muscle mass preservation, and an oxidative phenotype (21). The multiple mechanisms by which SCFAs influence athletic performance via modulation of skeletal muscle function is an area of growing research.

Population-based cohort studies have documented the correlation between the gut microbiota and both physical activity, particularly vigorous physical activity, and exercise (24–27). It is important to distinguish between “physical activity” and “exercise” (3) when considering the interaction between sport and the gut microbiota (Text Box 1). When assessing the effect of sport on the gut microbiota or the role of the gut microbiota in athlete health and performance, exercise is the more accurate classification. However, exercise has different modes and varying degrees of intensity, which may differentially impact the gut microbiota (23, 28–32). For instance, cardiorespiratory exercise induced immediate changes in the gut microbiota composition, while resistance exercise had no effect (23). This may be due to differences in the metabolic pathways involved in and activated by different exercise modalities (23).

Additionally, factors such as dietary intake, colonic transit time, training status, shared training environment, health or disease status, age, or gender may present confounding factors in assessing the bidirectional relation between sport and the gut microbiota (2, 33–35). Recent reviews have discussed dietary intake (2, 15) and supplements (17) on the gut microbiota of athletes. Numerous cross-sectional studies have reported the relation between exercise, athletes’ habitual diets, and the gut microbiota (36). Additional studies have investigated the impact of dietary and exercise interventions on the gut microbiota in rodents or in sedentary or otherwise unhealthy human populations (2). Importantly, there is research on the effects of combined exercise and dietary interventions in athletes (**Table 1**) (37–44).

**TABLE 1:** Combined diet, exercise, and gut microbiota interventions in athletes^1^

Study (reference)	Year	Diet/supplement	Exercise/test	Duration	Participants	Microbiota effects	Metabolite effects	Metabolic effects	Performance effects
Moreno-Pérez et al. (37)	2018	Protein supplement (10 g whey isolate + 10 g beef hydrosylate) vs. control (maltodextrin)	Habitual training (≥5× per week)	10 wk	24 males, ages 18–45 y, cross-country runners	↑ Bacteroidetes in protein group; ↓ *Roseburia, Blautia, Bif. longum* in protein group	↔ Fecal pH, water content, ammonia, SCFA concentration, plasma or urine malondialdehyde in protein group	—	—
Murtaza et al. (38)	2019	HCHO vs. PCHO vs. LCHF	Habitual training test: *V*O_2_peak, walking economy, 10-km race time, 25-km long walk time, respiratory exchange ratio, fuel oxidation rate	3 wk	21 males, ages 20–35 y, race walkers	↔ Enterotype, diversity (Shannon, Simpson, weighted/unweighted UniFrac) in diet groups; ↓ *Faecalibacterium* spp., *Bifidobacterium, Veillonella, Streptococcus, Succinivibrio, Odoribacter, Lachnospira* spp. in LCHF group; ↑ *Dorea* spp., *Bacteroides* spp., *Enterobacteriaceae, Peptostreptococcaceae, Barnesiellaceae, Akkermansia* in LCHF group; LCHF × enterotype interaction (↓ *Bif*., ↑ *Sutterella* in *Bacteroides* enterotype; ↑ Clostridiales in *Prevotella* enterotype); ↑ Clostridiales, *Ruminococcaceae, Coprococcus* spp., *Akkermansia muciniphila, Bifidobacterium, Streptococcus* in PCHO group; ↑ *Clostridiaceae, Lachnospiraceae, Ruminococcaceae, Streptophyta* in HCHO group	—	↑ Fat oxidation in LCHF group *Bacteroides* ∼ fat oxidation (–) in LCHF group	↓ Exercise economy, 10-km race performance in LCHF group; ↑ exercise economy, 10-km race performance in HCHO and PCHO groups; *Dorea* ∼ exercise economy (*–*) in LCHF group
Karl et al. (39)	2017	Rations (control) vs. rations + protein supplement (whey) vs. rations + carbohydrate supplement	4-d cross-country ski-march (STRESS)	4 d	73 soldiers, age >18 y, 71 men, 2 women	↑ α-Diversity (Shannon) post STRESS (no difference between groups); ↔ richness (Chao1, total observed OTUs); ↑ Firmicutes/Bacteroidetes ratio post STRESS (no difference between groups)	↑ p-Cresol post STRESS (no difference between groups)	↑ Sucralose and mannitol excretion post STRESS (no difference between groups); ↔ LPS	—
Son et al. (40)	2020	Probiotic supplement (1012 CFU each *Lactobacillus acidophilus, L. casei, L. helveticus, Bifidobacterium bifidum*) vs. placebo; divided into groups based on dietary intake: group 1 (high-protein, reduced fiber), group 2 (high-protein, adequate fiber), group 3 (adequate protein, restricted fiber), group 4 (sedentary control)	Habitual training	60 d	15 males, bodybuilders	↔ α-Diversity (Shannon, Simpson), probiotic bacteria (*Lactobacilli, Bifidobacterium*) in probiotic vs. placebo group; ↑ Paraprevotella in probiotic group; ↑ *Megamonas, Anaerostipes, Dorea* in placebo group; ↔ α-diversity in group 1 vs. group 4; ↑ no. of species, Chao1 richness, ACE, Jacknife in groups 2 and 3 vs. group 4; ↑ *Haemophilus, Streptococcus* in group 1; ↑ *Bifidobacterium* in group 2; ↑ *Faecalibacterium* in group 3	↔ SCFAs	—	—
Huang et al. (41)	2020	Probiotic supplement (3× 1010 CFU/d *L. plantarum* 128) vs. placebo	Habitual training test: $\dot{V}{{\rm{O}}_2}\max $, endurance performance	4 wk	20 males, triathletes	↑ *Akkermansia, Bifidobacterium, Butyricimonas, Lactobacilli* in Probiotic group; ↓ α-diversity (Shannon), *Anaerotruncus, Caproiciproducens, Coprobacillus, Desulfovibrio, Dielma*, Family_XIII, *Holdemania, Oxalobacter* in Probiotic group; ↔ Firmicutes/Bacteroidetes ratio	↑ SCFAs (acetate, propionate, butyrate) in probiotic group	↔ Body composition (bone, fat, lean %), blood biochemistry (glucose, lipids, creatinine, liver enzymes, blood cell counts)	↔ $\dot{V}{{\rm{O}}_2}\max $; ↑ endurance performance
Martarelli et al. (42)	2011	Probiotic supplement (109 CFU/d, 1:1 of *L. rhamnosus* IMC 501, *L. paracasei* IMC 502) vs. control (no supplement); all athletes on controlled diet developed based on athlete's basal metabolism, body composition, and energy expenditure (methods not described)	Controlled training developed based on athlete's basal metabolism, body composition and energy expenditure (methods not described)	4 wk	24 males, cyclists	↑ *Lactobacilli* in Probiotic group	—	↑ Reactive oxygen metabolites after physical activity in control group (not in probiotic group); ↑ plasma antioxidants in probiotic group	—
West et al. (43)	2011	Probiotic supplement (109 CFU/d *L. fermentum* PCC) vs. placebo	Habitual training test: $\dot{V}{{\rm{O}}_2}\max $, peak power output, postexercise cytokine response	11 wk	64 males, 35 females, cyclists	↑ *Lactobacilli* in males in probiotic group (not in females)—obtained from subset of 10 males and 10 females from each group	—	↓ Severity of GI and lower respiratory illness in males in probiotic vs. placebo; ↑ number and duration (↓ severity) of lower respiratory illness in females in probiotic vs. placebo; ↓ cytokine response to acute exercise in probiotic group; ↔ upper respiratory tract infection, mucosal immunity (lactoferrin, lysozyme, SIgA)	↔ $\dot{V}{{\rm{O}}_2}\max $
Axelrod et al. (44)	2020	Probiotic supplement (2× 108 CFU/d *L. salivarius* UCC118) vs. placebo	Test 2-h treadmill at 60% $\dot{V}{{\rm{O}}_2}\max $	4 wk	7 trained endurance athletes, ages 18–45 y	↓ Verrumicrobia, Verrumicrobiae, Verrumicrobiales, *Verrumicrobiaceae*, Prosthecobacter fusiformis in Probiotic group; ↔ diversity/richness (Chao1, ACE, Shannon, Simpson)	—	↓ GI permeability of sucrose in probiotic group; ↔ lactulose and rhamnose excretion, fecal zonulin, core temperature, IL-6	—

1 ACE, Abundance-based Coverage Estimator;*Bif*., *Bifidobacterium*; GI, gastrointestinal; HCHO, high carbohydrate; LCHF, ketogenic low-carbohydrate, high-fat; OTU, operational taxonomic unit; PCHO, periodized carbohydrate; SIgA, secretory immunoglobulin A; STRESS, 4-d cross-country ski march, $\dot{V}{{\rm{O}}_2}\max $, maximal oxygen uptake; *V*O_2_peak, peak oxygen uptake; ↑, significant increase; ↓, significant decrease; ↔, no significant difference.

## The Effect of Sport on the Gut

In addition to affecting the gut microbiota, exercise also impacts gastrointestinal physiology. Although exercise typically acts as a beneficial, or “hormetic,” stress, it can become detrimental if increased duration and intensity are not supported by adequate training, rest, nutrition, and antioxidant status (17). Exercise activates the autonomic nervous system, increasing circulating concentrations of cortisol and catecholamines, epinephrine, and norepinephrine, in peripheral tissues and the gastrointestinal tract (45). This results in reduced blood flow to the gastrointestinal tract, causing hypoxia, ATP depletion, and oxidative stress (46). These effects damage the gut barrier, increasing intestinal permeability, endotoxemia, nutrient depletion, and inflammation (46). The gastrointestinal tract responds to stress activation by releasing neurotransmitters such as γ-aminobutyric acid (GABA), neuropeptide Y, and dopamine, which are associated with gastrointestinal disturbances (45). These physiological effects are proportional to the intensity, duration, and frequency of exercise (45, 46).

While low- to moderate-intensity exercise promotes gastrointestinal motility and transit time, intense [>60% maximal oxygen uptake ($\dot{V}{{\rm{O}}_2}\max $)] or prolonged (≥2 h) exercise may have the opposite effect, as well as create acute gastrointestinal disturbances (45, 47, 48). Regular exercise promotes adaptations to maintain intestinal blood flow and reduce inflammation, although recovery must also be adequate (46). Gastrointestinal issues are common, particularly among endurance athletes, with 30–50% of athletes experiencing gastrointestinal distress symptoms (48). These symptoms may be caused by physiological, mechanical, psychological, and nutritional factors, including reduced blood flow, increased gut permeability, increased production of stress hormones and inflammatory cytokines, and inadequate gastric emptying (45, 47, 48). However, outside of endurance running, gastrointestinal symptoms are rarely assessed (49). For instance, a study in soldiers participating in a 4-d rigorous cross-country ski march revealed increased intestinal permeability but did not report gastrointestinal symptoms, making the implications on subjective experience and the impact on exercise performance unclear (39).

The gut epithelium has a high turnover rate (3–5 d) and requires large amounts of energy and nutrients (50). Athletes training at high intensities for long periods without adequate fueling are at risk for disturbances in gut integrity and function and gastrointestinal symptoms. In particular, inadequate habitual carbohydrate intake increases the proinflammatory stress response to prolonged, continuous strenuous exercise (47, 51). However, research has primarily focused on the effects of acute intake (before and during) on gastrointestinal symptoms during exercise rather than habitual diet, although an increasing number of athletes and researchers focus on food-avoidance strategies, such as a low fermentable oligo-, di-, monosaccharides and polyols (FODMAP) diet or gluten-free diet, as discussed below (47, 48).

The increased oxidative stress and disturbances to the gut barrier function that cause gastrointestinal symptoms also influence the gut microbiota (22, 45). Translocation of LPS, components of gram-negative bacteria resulting from increased gut permeability, causes endotoxemia and triggers proinflammatory cytokine secretion into the gastrointestinal tract that may influence the gut microbiota and further exacerbate the condition (22). Conversely, the microbial metabolites butyrate and propionate serve as energy sources for colonocytes, reducing mucosal degradation, gastrointestinal permeability, and inflammatory cytokines (22, 45). As alterations in microbial composition and diversity have been associated with gastrointestinal distress prevalence in athletes, the gut microbiota composition may be used as a biomarker for metabolic and systemic stress after exercise (22). For instance, a study investigating the acute effects of an exercise bout on the serum and fecal metabolome and the gut microbiota demonstrated that a single bout of exercise upregulated metabolic pathways of skeletal muscle substrate utilization and carbohydrate metabolites in serum, increased fecal ammonia and amino acid metabolites, and increased the abundance of *Clostridia* (52). Thus, acute changes in microbial and metabolite profiles may provide information regarding the effects of exercise on the gastrointestinal tract and metabolism. Furthermore, gastrointestinal symptom assessments could complement information on gut microbiota composition when considering the impact of exercise on the gut microbiota and the need for gut-centric dietary strategies.

## Dietary Strategies for Sport and the Gut

Diet is 1 tool that athletes use to optimize their fitness, performance, and recovery (1). Dietary strategies for sport seek to optimize training, performance, and recovery via supplementation of specific nutrients (e.g., protein, carbohydrate loading, iron), restriction of energy or certain food categories (e.g., low-FODMAP diet, gluten-free), and adequate hydration; however, the effects of these dietary strategies on the gut microbiota are not well understood (17, 53, 54). Alternatively, increasing research indicates that dietary strategies for improving gastrointestinal health (e.g., probiotics, prebiotics, and synbiotics) represent promising opportunities to optimize the interaction between the gut and sport, with the potential to enhance athletes’ health and performance. The following sections discuss the effects of dietary strategies on the gut microbiota and athletic performance.

### Protein

Protein is the main component of skeletal muscle. However, specific amino acids differ in their uptake and catabolism by the liver and skeletal muscle and their ability to regulate the muscle protein synthetic response (55). Essential amino acids, particularly branched-chain amino acids (BCAAs), are crucial for muscle protein synthesis and result in a greater muscle protein synthetic response than nonessential amino acids (55, 56). Therefore, dietary protein influences protein utilization and the anabolic response of skeletal muscle to exercise (55).

Although recommendations vary, athletes may need upwards of twice as much protein as the general population (1.2–1.7 vs. 0.8 g · kg^–1^ · d^–1^) to maintain protein synthesis, energy production, immune function, and gut integrity as a result of exercise-induced stress (54). This is true for endurance and resistance-trained athletes. Indeed, endurance athletes may need to ingest a higher amount of protein in the postexercise recovery period (∼0.5 vs. ∼0.3 g · kg^–1^ within 3–5 h of exercise), particularly if endurance exercise is performed in a fasted state, as this may increase myofilament proteolysis (57–59). Although variable based on dietary and physiological factors such as digestibility, quantity and composition of amino acids, the food matrix, and presence of other nutrients (60, 61), ∼10% of protein is not digested and may undergo proteolytic fermentation by bacterial proteases in the colon (62–64).

Concerning gut microbiota metabolism, amino acids can be classified by their fermentation metabolites: sulfur-containing amino acids, aromatic amino acids, and tryptophan (60). These metabolites include branched-chain fatty acids and SCFAs, ammonia, sulfides, indolic, and phenolic compounds (61, 64). While some of these metabolites (e.g., SCFAs and indole) may have beneficial effects like improving gut integrity, other metabolites (e.g., ammonia and p-cresol) decrease gut epithelium integrity (64, 65). Excess protein intake may lead to levels of proteolytic metabolite production that overwhelm the hosts’ ability to assimilate, transform, or detoxify harmful metabolites (61), contributing to adverse effects on intestinal barrier function, inflammation, and colonic health (60, 61, 63–66).

Protein supplements, including BCAAs and taurine added to energy drinks, are commonly used by athletes to enhance the anabolic and adaptive effects of exercise on skeletal muscle and improve recovery (67–69). Excess taurine leads to elevated taurocholic acid (TCA), deoxycholic acid (DCA), and hydrogen sulfide (H_2_S) concentrations, which are associated with increased risk of colorectal cancer (70); however, the effects of these supplements on the athletic gut microbiota are unclear (17, 54). To our knowledge, there is only one intervention study that has investigated the effects of protein supplements on the gut microbiota in athletes (37). In this study, male cross-country runners consumed a protein supplement (10 g whey isolate and 10 g beef hydrosylate) or a placebo (maltodextrin) for 10 wk. Protein powder consumption was associated with a decrease in *Lachnospiraceae, Roseburia, Blautia, Synergistales, Coprococcus, Lactobacillales, Bacilli*, and *Bifidobacterium longum*, as well as a higher abundance of Bacteroidetes and lower abundance of Firmicutes relative to the placebo. There were no differences between groups at baseline or after the intervention in α-diversity (Chao1, equitability, phylogenetic tree, number of observed species, Shannon index, Simpson index), β-diversity (unweighted UniFrac), or microbial metabolites (i.e., SCFAs, ammonia). Thus, protein supplementation influenced the abundance of specific taxa with limited effects on the community's diversity and function (i.e., metabolites).

Additional studies have investigated the effects of protein supplementation or high-protein diets in sedentary adults with overweight and obesity. McKenna et al. (71) investigated the effects of moderate (0.8–1.0 g · kg^–1^ · d^–1^) and high (1.6–1.8 g · kg^–1^ · d^–1^) beef protein consumption combined with resistance training in a cohort of healthy, overweight, middle-aged adults. In this study, participants in the high-protein group had decreased abundance of *Veillonellaceae, Akkermansia*, uncultured *Eggerthellaceae*, and *Ruminococcaceae* UCG-010 following 1 wk of dietary habituation relative to baseline (71). However, there were no differences between the moderate- and high-protein groups in strength gains in response to resistance training (71). Cronin et al. (72) investigated the effect of whey protein supplementation (24-g blend of whey concentrate, isolate, and hydrolysate) in sedentary adults with overweight and obesity with or without exercise on the gut microbiome and reported no effects of the protein supplement or exercise on microbiota composition or metabolic pathways. The lack of effect of the supplement on gut microbial composition may have been due to lack of dietary control or the relatively short duration of the intervention (8 wk) compared with habitual exercise and supplementation undertaken by athletes (65). However, the authors did report a shift in the gut virome in the protein-supplemented groups with and without exercise due to virus particles present in the supplement and increases in trimethylamine-N-oxide (TMAO) and phenylacetylglycine (PAG) in the protein-supplemented group without exercise.

TMAO, produced from carnitine, choline, and phosphatidylcholine, is metabolized by the gut microbiota to trimethylamine (TMA), which is then converted to TMAO in the liver (14). TMAO and PAG are associated with increased risk of cardiovascular disease and adverse outcomes in cardiovascular disease patients (73, 74) and are elevated in athletes versus sedentary controls, potentially resulting from increased protein intake (75). In contrast, the addition of exercise decreases TMAO (72). The associations between TMAO and disease may be confounded by or dependent on kidney function, the gut microbiota, and the flavin-containing monooxygenase 3 (FMO3) genotype (4, 76–78). Fish is rich in preformed TMAO and has the greatest impact on circulating TMAO concentrations; however, fish intake is associated with decreased risk of cardiovascular disease (76, 77). Additionally, habitual intake of red meat, containing the TMAO precursor carnitine, and acute feeding of phosphatidylcholine, the predominant form of choline in foods such as eggs, are not associated with increased circulating TMAO (76, 4, 78, 79). Ultimately, the connections between TMAO, lifestyle factors (diet and exercise), and disease are complex, and it is difficult to draw conclusions based on the current state of the science.

Beaumont et al. (80) investigated the effects of a high-protein diet (∼30% energy intake) using either casein or soy (both providing 15% of energy intake) in overweight individuals. They reported no shift in the gastrointestinal microbiota, perhaps due to variability in the protein sources consumed by participants outside of the supplements or other aspects of dietary intake. However, this study reported a shift in bacterial metabolism and metabolite profiles toward products of amino acid degradation, including a decrease in butyrate and increases in 2-methylbutyrate, phenylacetylglutamine, and indoxyl sulfate.

The results of these protein-supplementation studies indicate that, while protein supplements may impact the gut microbiota composition, they have a greater impact on microbial metabolites (81, 82). The decrease in butyrate, a key SCFA, and increase in proteolytic metabolites could be detrimental to gastrointestinal health. Therefore, protein supplementation in athletes should be further assessed to determine whether this population experiences the same metabolic effects and whether these changes are associated with increased gastrointestinal distress or inflammation and performance.

Cross-sectional studies examining the relation between dietary intake and the microbiota in athletes have reported inconsistent results. For instance, Clarke et al. (83) reported that protein intake was positively correlated with microbial diversity, while Jang et al. (84) reported a negative association between protein intake and microbial diversity. These contradictory findings may be due to the athletes’ fiber intake, as those in the study by Clarke et al. met recommended fiber intake requirements, while those in the study by Jang et al. did not (15). A follow-up study to Clarke et al. investigating the metabolomic and metagenomic signatures of athletes and sedentary controls reported an increase in microbial genes related to amino acid biosynthesis and carbohydrate metabolism, as well as an increase in amino acid metabolites (e.g., TMAO and PAG) and SCFAs, suggesting that fiber intake was sufficient to balance the increased protein intake (75). Indeed, it has been suggested that the fiber, calorie, and fat content of the diet may have significant impacts on the effects of protein amount and type on the gut microbiota and health (65, 85, 86).

Animal studies investigating the effects of different protein types, focusing primarily on the comparison between animal- versus plant-based proteins, have reported differential effects such as a higher abundance of *Lactobacilli* (∼5-fold) and the ratio of Firmicutes to Bacteroidetes and lower butyrate (−1.4-fold), SCFA-producing bacteria (e.g., *Bacteroides* and *Prevotella*), LPS-binding protein (∼ −2-fold), and transcription factor CD14 receptor (∼ −0.4-fold) with meat versus nonmeat proteins (87–90). LPS-binding protein binds to CD14 to mediate the activation of macrophages to produce inflammatory cytokines, serving as a proxy for inflammation (89). Thus, these results suggest that soy, the plant-based protein used in these studies, elicited a greater inflammatory response than animal proteins (88, 89). Dairy proteins appear to have an intermediate effect between meat and nonmeat proteins (88), although results may differ between whey and casein components (91). However, these studies of protein type have been conducted almost exclusively in rodents, primarily use isolated protein sources, and often use protein intakes above the recommended daily guidelines. Effects of protein sources such as beef on the gut microbiota have more consistent findings in animal models than humans, in which limited to no impact of protein type has been reported, highlighting the need for more studies in humans (90). To our knowledge, only 1 study has investigated the effects of different protein types on the gut microbiota in humans; however, this study also added a high– or low–saturated fat component to the study design and reported that saturated fat consumption masked the effects of protein type (85). This again demonstrates the need to account for the intake of other dietary components (e.g., fat) in addition to protein.

Evidence suggests that the gut microbiota contributes to protein absorption and utilization (92) as well as skeletal muscle anabolism and functionality (gut–muscle axis) via fuel availability and storage and modulation of inflammation (19, 20, 93, 94). For example, probiotic supplementation (*Lactobacillus paracasei*) enhanced the bioavailability of plant proteins, elevating essential amino acid and BCAA concentrations to comparable concentrations of animal proteins (95). Additionally, when co-administered with protein, the probiotic *Bacillus coagulans* (GBI-30,6086) decreased epithelial cell inflammation, improved nutrient absorption, and produced proteases that increase amino acid absorption in humans (92). These effects may reduce muscle damage and boost muscle recovery, thereby enhancing adaptation and performance (92). Increasing the bioavailability and absorption of dietary protein and increasing muscle protein synthesis is 1 mechanism by which the gut microbiota may influence muscle mass and function. These effects may be partially regulated by SCFA production, affecting insulin sensitivity, inflammation, and release of insulin-like growth factor I (IGF-I) that modulate the balance between anabolic and catabolic processes (93, 96). Therefore, the gut–muscle axis may mediate the positive effects of exercise and diet on muscle anabolism and play a role in the age-related decline in muscle mass (i.e., sarcopenia) and disease-related muscle wasting (19, 20, 93, 94, 96, 97). For instance, increased abundances of *Oscillospira* and *Ruminoccocus* and decreased abundances of *Barnesiellaceae* and *Christenellaceae* helped accurately predict individuals with physical frailty and sarcopenia (97). However, due to the small sample size, it is unclear if differences in body composition, diet, and physical activity contributed to these differences in muscle function independently of the gut microbiota. However, alterations in the gut microbiota have been associated with phenomena including “anabolic resistance” that contribute to the development of sarcopenia (96). Therefore, growing research suggests that the gut microbiota plays a role in muscle function and anabolism via modulation of protein metabolism.

An additional area of interest is the effects of whole-food protein versus protein supplements as whole foods have been shown to have equal or superior ergogenic effects (1, 98–100). For instance, ingestion of whole eggs versus egg whites and whole milk versus fat-free milk result in greater amino acid uptake and postexercise myofibrillar protein synthesis (100, 101), suggesting that nonprotein components (e.g., lipids, carbohydrates, micronutrients, and other bioactive compounds) contribute to the postexercise protein synthetic response. The food matrix may also contribute to differential effects of whole-food protein sources on the gut microbiota, as the same quantity of protein in supplement form and the change in the amino acid profile as a result of protein isolation impact the protein digestion and absorption (60, 61, 98). For instance, purified proteins are digested more efficiently than protein-rich foods consumed in a mixed meal, which may decrease the amount of protein delivered to the large intestine, although the amount of purified protein ingested likely also influences colonic availability (60). It is unclear to what extent these differences in digestibility of protein types, and potential modulating effects of gut transit time, affect the athlete gut microbiota, health, and performance.

In summary, high-protein diets and protein supplements appear to have limited effects on the gut microbiota composition but shift the metabolite profile to greater production of proteolytic metabolites. This may lead to detrimental effects on gastrointestinal health and exacerbate exercise stress–induced symptoms of gastrointestinal distress in athletes, which may impair training and performance. However, these effects may be specific to the protein supplement type and depend on concomitant carbohydrate or fiber intake. Furthermore, the gut microbiota may also contribute to muscle protein anabolism and function throughout the lifespan via modulation of protein absorption and utilization.

### Fat

Intramuscular triglycerides and adipose tissue provide important fuel substrates for athletes during exercise (102, 103). Additionally, dietary fat modulates the gut microbiota composition and subsequently impacts metabolic health (104). The amount and type of dietary fat are important aspects of dietary quality and are important considerations for both athletic performance (102, 103, 105) and the health of the gut microbiota (104, 106–108).

Dietary fat intake is variable based on sport modality, training level, and body-composition goals (84, 102). Pre-exercise meals or snacks are generally low in fat to facilitate gastric emptying and minimize gastrointestinal distress during exercise (109). Conversely, there is interest in high-fat, low-carbohydrate ketogenic diets for athletes for performance enhancement or weight control (110, 111). However, while a high-fat, low-carbohydrate diet does enhance fat oxidation, there is no evidence to support the notion that it increases performance; instead, there is evidence that it may decrease exercise performance at higher intensities (102, 103, 110, 112). Alternatively, supplementation of omega-3 (ɷ-3) essential fatty acids may positively affect exercise performance via improved endurance capacity, recovery, and immune modulation (105). However, most studies have been conducted in untrained, amateur populations, and few focus on performance as an outcome, limiting the ability to determine their ergogenic effects in athletes (105).

Concerning the gut microbiota, research on fat intake has primarily centered on the effects of a high-fat, particularly high-saturated-fat, Western-style diet (104, 107, 113, 114). These studies reveal that the Western-style dietary pattern is associated with an increased Firmicutes to Bacteroidetes ratio and increased abundance of Proteobacteria, Mollicutes, and *Bilophila wadsworthia*, as well as a decrease in *Akkermansia muciniphila, Bifidobacterium* spp., and butyrate-producing taxa (62, 104, 113).

Additionally, a high-fat diet with concomitant restriction of carbohydrates, as in a ketogenic diet, may have differential effects on the gut microbiota and inflammation compared with a high-fat diet without carbohydrate restriction due to ketone body production (115). However, there are conflicting results regarding the effects of the ketogenic diet on gut microbiota composition, although evidence suggests that the gut microbiota mediates some of the beneficial effects of the ketogenic diet on neurological outcomes (116). In men with overweight and obesity, consumption of a ketogenic diet decreased *Bifidobacterium* and *Lactobacilli* and increased *Fusobacteria* and *Escherichia* (115). To our knowledge, only 1 study has investigated the effects of a ketogenic diet, compared with either a high-carbohydrate or periodized carbohydrate diet, on the gut microbiota of athletes (elite race walkers). The authors reported an increase in *Bacteroides* and *Dorea* and a reduction in *Faecalibacterium*, a known butyrate producer (38). Additionally, the abundance of *Bacteroides* and *Dorea* following the intervention was negatively associated with fat oxidation and exercise economy, respectively, suggesting a negative correlation of these taxa with exercise performance (38). Furthermore, recent reviews indicate that ketone supplementation does not benefit athletic performance, cognition, or muscle recovery in athletes and may induce gastrointestinal symptoms (117, 118).

In addition to the amount, the type of fat modulates the gut microbiota and downstream inflammatory signaling, which may have implications for athletic performance. While acute inflammation in response to exercise is necessary for the adaptive response and functional recovery of muscle, chronic or excessive inflammation can lead to detrimental effects such as reduced muscle strength and mass (93, 119). Different types of fat are associated with varying effects on the gut microbiota and consequential effects on inflammation (107, 114, 120). Saturated fat intake is associated with decreased microbiota diversity and richness in humans and increased availability and transport of LPS, leading to proinflammatory Toll-like receptor (TLR) activation in preclinical models (107, 121). Monounsaturated fat intake is also associated with decreased total bacterial numbers in humans and increased LPS in preclinical models but still leads to lower inflammation than saturated fat (121). However, polyunsaturated fat has no effect on diversity or richness in humans and increases the abundance of *Bifidobacterium, Lactobacilli*, and *Akkermansia muciniphila*, which are also increased by exercise (2, 107). ɷ-3 PUFAs increase SCFAs, improve gastrointestinal integrity and inflammation, and potentially affect communication along the gut–brain axis (108). Therefore, beneficial effects of ɷ-3 fatty acids on the gut microbiota may mimic the effects of exercise and contribute to health and performance benefits by promoting an anti-inflammatory bacterial profile and production of SCFAs. Conversely, the proinflammatory effects of high saturated fat intake on the gut microbiota may impair exercise-induced performance benefits on muscle anabolism.

Bile acids may also mediate some of the disparate effects of different dietary fats on lipid and carbohydrate metabolism, energy expenditure, and inflammation via the farnesoid X receptor (FXR) and G protein–coupled membrane receptor 5 (TGR5) (106, 121–123). Interactions of bile acids with these receptors also increase energy expenditure in skeletal muscle and decrease muscle fat deposition, suggesting that microbiota-mediated changes in the bile acid pool may influence skeletal muscle function (94, 124). Increased intramuscular triglycerides (IMTGs) have been reported in individuals with obesity and associated with insulin resistance, although athletes exhibit similarly high concentrations of IMTGs that can be used as fuel during exercise (125). It is now thought that the association with insulin resistance is due to increased intramuscular lipid metabolite concentrations, not IMTGs, and that accumulation of these metabolites is prevented by high IMTG turnover with exercise (125). Secondary bile acids produced by the gut microbiota also interact with FXR and TGR5 receptors and increase mitochondrial oxidative phosphorylation and fatty acid β-oxidation, which may have performance benefits such as better oxygen uptake, energy availability, and fatigue resistance (126). It is unclear whether bile acid modulation of IMTG content or mitochondrial function influence exercise capacity in athletes and, if so, how to optimize the concentration and composition of bile acids and secondary bile acids via type and amount of fat intake.

In summary, high fat, particularly high saturated fat, intake is linked to a proinflammatory microbiota composition with a reduced capacity to produce SCFAs and may induce gastrointestinal permeability, both of which can adversely impact performance. Conversely, ɷ-3 fatty acids may promote a beneficial microbiota profile, increased SCFAs, and reduced gastrointestinal permeability. However, current research on the ergogenic effects of ɷ-3 fatty acids is inconclusive (127).

### Carbohydrate and fiber

Highly digestible and readily absorbed carbohydrates are of great interest for sport. However, nondigestible carbohydrates (i.e., fibers and resistant starches) are of greater interest when considering the gut microbiota.

Carbohydrates function as one of the primary fuel sources during exercise (128). Dietary recommendations for athletes suggest high intakes of simple carbohydrates to maintain glucose homeostasis and low fiber intake before exercise to reduce gastrointestinal distress, also adding that plant-based high-fiber diets may reduce energy availability (17, 109). Ingestion of simple carbohydrates (e.g., glucose, fructose, sucrose, dextrose) before and during exercise can reduce fatigue, improve performance, and promote water reabsorption and maintenance of euhydration (45, 129, 130). However, glucose and fructose load and the fructose-to-glucose ratio affect gut microbial fermentation and gastrointestinal distress (131). Ingesting fructose and glucose in equal quantities optimizes fructose absorption (132, 133) and reduces microbial fermentation, potentially reducing gastrointestinal distress symptoms. Lactose may also serve as a good fuel source before, during, and after exercise for increased performance and recovery while also potentially promoting beneficial effects on the gut microbiota, such as increases in *Bifidobacteria* and *Lactobacilli* (134).

Carbohydrate loading is also a common strategy used by endurance athletes to maximize glycogen concentrations before a competition (135). The goal of carbohydrate loading is to maximize carbohydrate absorption and glycogen storage. Thus, carbohydrates that will not be digested and absorbed in the small intestine, like fiber and resistant starch, are generally avoided. Interestingly, ingestion of potatoes during cycling is as effective as carbohydrate gels to support performance, despite having a much higher fiber content (11.2 vs. 2.3 g) (136). However, gastrointestinal symptoms (abdominal pain, bloating, and discomfort) were higher in the potato group, limiting the use of such practices among athletes.

Athletes focused on maximizing glycogen storage may ingest high amounts of carbohydrates but avoid nondigestible carbohydrates (45, 137). Evidence suggests that a high-carbohydrate, low-fiber dietary pattern has detrimental effects on intestinal health and microbes, including altered intestinal transit times, loss of bacterial diversity, and reduced SCFA production (11, 138, 139). There is a positive association between total dietary fiber per kilocalorie energy and the abundance of *Bifidobacterium* (140). Furthermore, adequate intake of nondigestible carbohydrates may also negate the potentially adverse effects of microbial proteolytic fermentation and its metabolites as nondigestible carbohydrates are preferentially metabolized by the gastrointestinal microbiota (60, 64, 65). Indeed, bodybuilders with high protein and restricted dietary fiber intake had greater microbiota similarity to sedentary controls (i.e., reduced α-diversity) compared with bodybuilders with adequate fiber intake (40). These microbiota characteristics may adversely affect long-term health and induce short-term gastrointestinal distress in athletes. This makes it even more important for athletes consuming high-protein diets to ensure adequate intake of nondigestible carbohydrates to prevent gastrointestinal distress and inflammation (45). Since athletes typically have increased energy intake relative to sedentary individuals (83), fiber intake should be scaled appropriately. Ultimately, athletes should strive for adequate fiber intake (14 g/1000 kcal) to promote gastrointestinal health and athletic performance, although avoidance directly before or after exercise may be warranted due to the potential for gastrointestinal distress.

SCFAs are linked to muscle function and glycogen accretion in skeletal muscle (19, 20). Therefore, reduced SCFAs due to a low-fiber diet may affect exercise capacity and performance. Studies in mice by Donatto et al. (141) (oat bran containing β-glucan, 300 g/kg chow) and Okamoto et al. (142) (hemicellulose and lignin, 14.6% neutral detergent fiber) revealed that nondigestible carbohydrate supplementation with exercise, either swimming or treadmill running, respectively, increases muscle glycogen concentration, SCFA production, and time to exhaustion while decreasing the postexercise inflammatory response. While muscle glycogen content is well correlated with endurance performance (143), the effect of increased SCFA production and systemic availability (18) on athletic performance in humans is unclear. Okamoto et al. (142) reported that infusion with acetate improved endurance exercise capacity in antibiotic-treated mice while Scheiman et al. (144) reported increased performance with propionate and *Veillonella atypica*, which converts lactate to propionate, inoculation in mice. The mechanism(s) of these ergogenic effects may involve increased glycogen or glucose fuel availability (19), increased water reabsorption (145), or direct utilization of metabolites (e.g., propionate) (144). Fiber intake and SCFAs may also decrease gastrointestinal permeability (146) and influence the immune response and inflammation via interaction with the gut-associated lymphoid tissue (GALT) (147). A study on the effects of butyrylated high-amylose maize starch in healthy adult cyclists increased butyrate and propionate concentrations, increased *Parabacteroides distasonis* and *Faecalibacterium prausnitzii*, and maintained IL-10 concentrations (an anti-inflammatory cytokine) (148). Another study on the effects of a low-dose (6 g/d), partially hydrolyzed guar gum fiber on the gut microbiota and recovery in athletes revealed increased Actinobacteria, decreased Bacteroidetes and *Clostridium cluster XI*, fecal defecation characteristic improvements, and reduced diarrhea (149), thus having a potential indirect effect on performance.

### Prebiotics

A prebiotic is “a substrate that is selectively utilized by host microorganisms conferring a health benefit” (150). While many fibers have prebiotic effects and are considered candidate prebiotics (e.g., resistant starch; polydextrose; β-glucans; pectin; soy-, xylo-, arabinoxylo-, and malto-oligosaccharides) (150–152), only fructo-oligosaccharides (present in artichokes, asparagus, bananas, chicory root, garlic, onions, leeks, wheat) (11) and galacto-oligosaccharides (derived from lactose) (153) are readily accepted as prebiotics (150). The health benefits of prebiotics include gastrointestinal health (e.g., pathogen inhibition), mental health (e.g., energy and cognition), and bone health (e.g., mineral absorption), all of which play important roles in the health and performance of athletes (150, 154).

While increasing prebiotic intake may decrease effective carbohydrate intake and glycogen storage, it has been postulated that microbial production of SCFAs from prebiotic fermentation may improve glycogen storage and metabolism (19, 155). To our knowledge, no studies have investigated the effects of prebiotic supplementation alone on exercise performance in athletes (156) (**Table 2**) (141, 142, 157–161). However, a study in asthmatic adults with hyperpnea-induced bronchoconstriction, a surrogate for exercise-induced bronchoconstriction, demonstrated that galacto-oligosaccharide supplementation (5.5 g/d) improved exercise-induced bronchoconstriction and reduced inflammation (157). Another study investigated the effect of exercise training in combination with inulin-propionate ester (IPE) supplementation in women with overweight and reported that IPE increased fat oxidation compared with a placebo (158). However, IPE has distinct effects compared with inulin alone on the gut microbiota and metabolome (162), making it difficult to determine whether the observed effects were due to inulin's prebiotic capacity or the esterified propionate.

**TABLE 2:** Prebiotic or synbiotic supplementation with exercise^1^

Study (reference)	Year	Prebiotic/synbiotic	Exercise/test	Duration	Subjects	Results
Prebiotics						
Donatto et al. (141)	2010	Oat bran (300 g/kg chow)	Swimming	8 wk	Rats (Wistar, male, 2 mo old)	↑ Time to exhaustion, hepatic glycogen in oat bran group; ↓ IL-6, IL-10, corticosterone in oat bran group
Okamoto et al. (142)	2019	LMC (cellulose) vs. HMC (cellulose, hemicellulose, lignin) vs. antibiotics vs. control	Forced-treadmill running	6 wk	Mice (C57BL/6J, male, 10 wk old)	↓ Time to exhaustion, muscle mass in LMC and antibiotic groups; ↑ time to exhaustion in antibiotic mice with acetate infusion (not with butyrate); ↔ body mass, muscle mass in antibiotic mice with acetate infusion and in LMC mice with HMC fecal transplant + inulin; ↑ time to exhaustion, SCFAs in LMC mice with HMC fecal transplant + inulin; ↑ white adipose tissue mass in LMC group; ↔ body mass gain, dietary intake; ↓ SCFAs (fecal and plasma) in LMC and antibiotic groups; ↑ Firmicutes/Bacteroidetes, Actinobacteria, *Lactococcus, Allobaculum* in LMC group; ↓ Shannon diversity, *Prevotella* in LMC group
Williams et al. (157)	2016	B-GOS (5.5 g/d) vs. placebo	EVH	3 wk	10 adults (with asthma and HIB, 5 males, 5 females) and 8 adult controls (5 males, 3 females)	↓ Peak post-EVH fall in pulmonary function following B-GOS; ↓ airway inflammation (chemokine CC ligand 17, TNF-α) following B-GOS in HIB group; ↓ CRP following B-GOS in HIB and control groups
Malkova et al. (158)	2020	IPE (10 g/d) vs. placebo	Supervised endurance exercise, submaximal *V*O_2_ treadmill test	4 wk	20 adults (overweight women)	↑ Fat oxidation in IPE group
Synbiotics						
West et al. (159)	2012	Gut Balance (synbiotic; *Lactobacillus paracasei* 431, *Bifidobacterium animalis* ssp*. lactis* BB-12, *L. acidophilus* LA-5, *L. rhamnosus* LGG, raftiline, raftilose, lactoferrin, immunoglobulins, acacia gum) vs. acacia gum (prebiotic)	Habitual exercise (cycling)	3 wk	22 adults (healthy males)	↑ *L. paracasei* in synbiotic vs. prebiotic group; ↓ increase in IL-16 in synbiotic vs. prebiotic group; ↔ fecal SCFAs, immunity, GI permeability in both groups
Coman et al. (160)	2017	Fermented milk (synbiotic; *L. rhamnosus* IMC 501, *L. paracasei* IMC 502, oat bran fiber) vs. control (fermented milk)	Habitual exercise (intense gym-training program)	4 wk	10 adults (healthy, 3 males, 7 females)	↑ *Lactobacilli* spp., *Bifidobacterium* spp., secretory IgA, improvement in intestinal regularity, ease of defecation, and improved upper respiratory symptoms in synbiotic group; ↓ Lipid hydroperoxide in synbiotic group
Roberts et al. (161)	2016	Pro/prebiotic/antioxidant (*L. acidophilus* CUL-60, *L. acidophilus* CUL-21, *Bif. bifidum* CUL-20, *Bif. animalus* ssp*. lactis* CUL-34, fructo-oligosaccharides, α-lipoic acid, N-acetyl-carnitine) vs. pro/prebiotic vs. placebo	Long-distance triathlon	12 wk pre-race, 6 d post-race	30 adults (healthy, recreational athletes, 25 males, 5 females)	↓ Endotoxin pre- and post-race with pro/prebiotic/antioxidant, post-race with pro/prebiotic; ↑ gastrointestinal permeability with placebo; ↔ mean race time

1 B-GOS, bimuno-galacto-oligosaccharide; *Bif*.,*Bifidobacterium*; CRP, C-reactive protein; EVH, eucapnic voluntary hyperpnea; GI, gastrointestinal; HIB, hyperpnea-induced bronchoconstriction; HMC, high microbiome-accessible carbohydrate; IPE, inulin-propionate ester; LMC, low microbiome-accessible carbohydrate; ↑, significant increase; ↓, significant decrease; ↔, no significant difference.

### Probiotics

Probiotic supplementation is a topic of interest among athletes to increase health and performance (36, 156, 163–170). Probiotics are “live microorganisms that, when administered in adequate amounts, confer a health benefit on the host” (171). Conventional probiotics include *Bifidobacterium* spp. and *Lactobacilli*, although other bacteria investigated in athletes include *Bacillus* spp., *Enterococcus* spp., *Streptococcus* spp., *Veillonella*, or the yeast *Saccharomyces boulardii*, which have been reviewed elsewhere (2, 36, 156, 163–170, 172).

Briefly, probiotics reduce infection, inflammation, muscle soreness, and gastrointestinal permeability or distress. Thus far, the most substantive evidence for probiotic benefits is improvements in the incidence, duration, and severity of upper respiratory tract infections (2, 163), which may indirectly improve athletic performance (156). The studies reporting improvement in respiratory symptoms include organisms from the *Lactobacilli* family (172, 173). *L. salivarius*, may also reduce gastrointestinal permeability via increases in butyrate-producing taxa *Roseburia* and *Lachnospiraceae* and decreases in *Verrumicrobia* (44). While there is evidence of shared mechanisms of probiotic functions, the benefits of probiotics are often dependent on the strain and dose of the probiotic (163, 164). The majority of studies reporting positive effects on gastrointestinal barrier function use multi-strain formulations (172). Probiotics may attenuate the effects of intense exercise on gastrointestinal distress and muscle soreness in athletes by improving intestinal permeability and antioxidant status and reducing inflammation (36, 156, 163, 171, 174, 175), potentially via interaction with GALT (176). For example, a daily multi-strain probiotic (Ultrabiotic 60^TM^, Bioceuticals, Australia AustL# 259813) containing 10 different strains from the genera *Lactobacillus, Bifidobacterium*, and *Streptococcus* and SBFloractiv™ (Bioceuticals, Australia AustL# 285024) containing the yeast *Saccharomyces boulardii* decreased muscle soreness in elite rugby athletes (175). While there was no main effect of treatment on inflammation, muscle soreness was positively correlated with salivary C-reactive protein (CRP) and negatively correlated with motivation and sleep quantity and quality (175). A combination of *L. rhamnosus* and *L. paracasei* increased plasma antioxidants and mitigated the exercise-induced rise in reactive oxygen species (ROS) while also increasing *Lactobacillus* in participants (42). Of interest to athletes while traveling, *Saccharomyces boulardii* and a combination of *L. acidophilus* and *B. bifidum* help prevent traveler's diarrhea (177).

Probiotics may also improve nutrient absorption and utilization, glycogen storage, body composition, energy harvest, hormone production, and cognition and mood via mechanisms such as bioactive metabolite production (e.g., SCFAs, neurotransmitters), modulation of gut pH, and alterations in the gut microbiota activities (36, 92, 163, 164, 169, 178, 179). For instance, *L. plantarum* increased endurance performance in triathletes concurrent with an increase in fecal SCFAs (41). A study in mice revealed that a bacterial strain isolated from an Olympic weightlifting athlete [*L. salivarius* subsp. *salicinius* (SA-03)] improved endurance performance and muscle strength via increased hepatic and muscular glycogen and decreased lactate, blood urea nitrogen, ammonia, and creatine kinase after exercise (178). However, more studies show ergogenic effects of multi-strain probiotics than single-strain probiotics (163), suggesting that multiple strains may act in a complementary way to provide performance benefits. Probiotics may, therefore, benefit athletic performance via both direct and indirect mechanisms, although the evidence of ergogenic effects remains scarce (156, 163, 166).

Differences in strains and doses of probiotics and individuals’ baseline diet, immune status, and microbiota composition may contribute to variability in findings between studies, making comparisons and conclusions difficult (2, 36, 40, 168). Most probiotic supplementation studies in athletes do not assess the gut microbiota, making it difficult to determine whether efficacy depends on baseline or changes in the participants’ gut microbiota composition (2, 166). Concurrent dietary intake, particularly intake of fiber and prebiotic substrates, may also impact the probiotic effects and should therefore be accounted for in analyses (180). This is important as consumers should be aware that probiotic supplementation alone may not have the intended effects if not supported by a diet with adequate nutrition. Additionally, probiotic supplementation studies in athletes typically have small sample sizes (i.e., 10 to 30 participants) and often include only or predominantly male participants (167), often the case in sports and exercise research (181), but which is problematic because there may be gender-specific effects (43). For example, in West et al. (43), probiotic supplementation with *Lactobacillus fermentum* (PCC^®^, Probiomics Ltd, Sydney, Australia) decreased gastrointestinal symptoms in males but increased the incidence and duration of symptoms in females.

There is increasing interest in the effect of live cultures in fermented foods (171, 182), and their effects or association with the gut microbiota (183). However, few studies have investigated the effects of fermented foods, including yogurt, kefir, sauerkraut, on exercise (184–187). Three studies using kefir or fermented milk reported decreased exercise-induced CRP or creatine phosphokinase and muscle soreness, indicating a positive effect of these fermented foods on reducing inflammation (185–187). One study in mice reported an ergogenic effect of kefir on strength and endurance (184). Therefore, fermented foods containing live microorganisms may confer beneficial effects on inflammation and exercise performance.

### Synbiotics

A synbiotic is “a mixture comprising live microorganisms and substrate(s) selectively utilized by host microorganisms that confers a health benefit on the host” (180). A synbiotic may be a combination of a probiotic and a prebiotic (complementary synbiotic), although the individual components do not necessarily need to meet the criteria for pro- and prebiotics as long as they act synergistically when co-administered (synergistic synbiotic) (180). Thus, the prebiotic component may enhance the functionality of the probiotic (synergistic synbiotic), or the 2 components may provide independent beneficial functions upon introduction to the gut and its resident microbes (complementary synbiotic) (180). This combination of microorganisms and selectively utilized substrates (159–161) may have different effects than either prebiotic or probiotic supplementation alone (Table 2). However, to our knowledge, only 1 study has investigated the synergistic and independent effects of these components in physically active humans (159). West et al. (159) reported that synbiotic supplementation (*Lactobacillus paracasei* 431, *Bifidobacterium animalis* subsp.*lactis* BB-12, *L. acidophilus* LA-5, *L. rhamnosus* LGG, raftiline, raftilose, lactoferrin, immunoglobulins, acacia gum) was associated with a smaller increase in serum IL-16 concentrations compared with prebiotic (acacia gum) supplementation alone, but neither synbiotic supplementation nor acacia gum alone influenced SCFA concentrations, immunity, or gastrointestinal permeability. Therefore, synbiotics may have different or additional effects on athlete health and performance than prebiotic or probiotic supplementation alone.

### Micronutrients

Micronutrients contribute to immune function, inflammation, energy metabolism, and bone health, impacting athletic performance (51, 188–190). Adequate intakes of iron, zinc and vitamins A, E, C, B-6, and B-12 are essential for proper immune function, which may be compromised under conditions of heavy training and competition in athletes (51). Furthermore, dietary requirements for some micronutrients may be increased in athletes due to losses in sweat and urine and increased oxidative stress (51, 188). Additionally, female athletes are at higher risk of iron deficiency, compromising health and performance (191).

Micronutrient deficiencies can also impact the gut microbiota (192). Lack of antioxidant micronutrients (e.g., vitamins C and E and selenium) decrease the abundance of commensal gut bacteria while promoting an increase in *Escherichia coli* (192). In animals under increased stress conditions, an antioxidant blend of vitamin C, vitamin E, polyphenols, lipoic acid, and microbial antioxidants restored intestinal redox status, which was correlated with increased *Bifidobacterium* and *Lactobacilli* and decreased *E. coli* (193). However, excessive intake of some micronutrients may also increase infection susceptibility (51). For example, excessive iron supplementation in infants increases pathogenic microbes, including *E. coli*, and contributes to intestinal inflammation (194, 195). Thus, micronutrient supplementation under conditions of increased stress or micronutrient deficiency may have microbiota-mediated benefits on immunity and inflammation.

Calcium and vitamin D support bone health. Additionally, vitamin D may impact skeletal muscle mass and strength via regulation of calcium-dependent contraction, protein-dependent skeletal muscle anabolism, mitochondrial function, and insulin sensitivity (196, 197). Increases in *Bifidobacterium, Lachnospiraceae*, and *Bacteroides* in response to fiber intake are positively correlated with increased calcium absorption (195, 198). This may be due to SCFA production, which increases calcium absorption by lowered colonic pH or regulation of signaling pathways or gene expression (199). Vitamin D intake also impacts the gut microbiota, although variability in results precludes the ability to determine the effect of supplementation on specific taxa (200). The bidirectional relation between intake calcium and vitamin D and the gut microbiota has important implications for bone health (201) in athletes of all ages, whether for growth or maintenance of bone density, to reduce the risk of fractures.

### Food avoidance

Gastrointestinal issues are common among athletes. To alleviate symptoms, athletes may avoid or restrict certain foods that trigger symptoms. Athletes may also adopt nutritional strategies to increase gastric emptying and improve absorption of water and nutrients, including avoidance of high-FODMAP foods and gluten-containing foods (202).

FODMAP are nondigestible short-chain carbohydrates that increase the osmotic load within the gastrointestinal tract. Intestinal microbes can ferment these dietary components to form gas, which results in bloating and gastrointestinal distress in certain individuals (203). A recent study investigating FODMAP intake in endurance athletes reported high intake, both habitually and surrounding exercise, contributing to gastrointestinal symptoms (204). Preliminary results indicate that a low-FODMAP diet alleviates gastrointestinal symptoms in athletes (203, 205, 206). However, FODMAP also act as fuel for the gut microbiota, and their restriction may impact the composition and function of the community (207).

It has been postulated that it is the reduction in FODMAP foods on the gluten-free diet that may be affecting improvement in gastrointestinal symptoms rather than gluten itself (208–210). To our knowledge, only 1 study has investigated the effects of a gluten-free diet in nonceliac endurance athletes (211), which reported no effect of the gluten-free diet on performance, gastrointestinal symptoms, well-being, intestinal injury, or inflammatory markers relative to a gluten-containing diet. However, this was a small study (*n* = 13) with a short duration (7 d) and did not assess effects on the gut microbiota, limiting its ability to draw conclusions on effects for the general athlete population or assess potential long-term effects on health or the gut microbiota.

### Energy intake

Food avoidance may also be applied more generally to energy restriction to achieve a particular physique or weight for sport. This is prevalent among female athletes and may result in inadequate energy availability, menstrual dysfunction, and decreased bone mineral density termed the “female athlete triad” (212). Energy deficiency contributes to gastrointestinal distress in athletes (213). At the extreme, anorexia decreases gut microbiota diversity and richness and increases *Methanobrevibacter smithii*, Proteobacteria, and the ratio of Bacteroidetes to Firmicutes (214). Anorexia is also associated with an altered metabolomic profile, including reduced SCFAs (215). These differences in the microbiota and metabolomic profiles may contribute to the clinical manifestations of inadequate energy availability, including gastrointestinal symptoms and compromised bone density (214). Prebiotic and probiotic supplementation and SCFAs have shown promising effects in the maintenance of and improvement in bone density and bone resorption, potentially via increased calcium absorption or IGF-I (214, 216). Therefore, microbiota-targeted therapies may complement dietary and psychiatric treatments for athletes with inadequate energy intake and/or disordered eating.

On the other side of the spectrum, many athletes have increased energy intake relative to sedentary controls (83). Much of this energy is utilized to support the energy demands of exercise, muscle remodeling and repair, and the health of the brain and immune system. However, greater quantities of food intake result in greater amounts of substrates being delivered to the large intestine due to the general efficiency of digestion and absorption. Each day, ∼15% of carbohydrates, 10% of protein, and 7% of fat escape digestion and are available for microbial fermentation (217). Maldigestion and malabsorption of nutrients may also be exacerbated by decreased blood flow and oxygen delivery (i.e., hypoxia) to the gut during exercise, causing changes in absorption, gut motility, and transit time (218). Intestinal hypoxia may also alter the mucosal-associated gut microbiota composition and disturb the balance of metabolic functions within this niche, potentially compounding the effects of these changes in gastrointestinal physiology on maldigestion and malabsorption. Increased caloric intake, independent of macronutrient composition changes, increases Firmicutes and decreases Bacteroidetes and microbiota diversity (219). Total calorie intake is positively correlated with the abundance of circulating serum zonulin, a marker of gastrointestinal permeability, in a large cohort of women, including athletes, anorexia nervosa patients, and normal-weight, overweight, and individuals with obesity (220). Zonulin was also negatively correlated with *Ruminococcaceae* and *Faecalibacterium*, both of which are butyrate-producing taxa, suggesting alterations in the gut microbiota composition (220). There were no differences in zonulin or gut microbiota composition detected between athletes and nonathletes, but differences in dietary intakes between the groups were not discussed, and therefore it is unclear whether disparities in dietary intake, or lack thereof, may have contributed to this homogeneity. Thus, while higher energy intake may contribute to differences in gastrointestinal function and the microbiota, athletes should obtain adequate dietary intake to support increased energy demands.

### Hydration

Hydration status is crucial for athlete health and performance and is supported by water and electrolyte transport across the gastrointestinal barrier. There is limited information on the effects of hydration status on the gut microbiota. However, lubiprostone, a clinical agent that is used to stimulate Cl^–^ secretion and thus cause water and electrolyte secretion in the gut, alters in the intestinal mucus layer and increases *Lactobacilli* in mice (221, 222). Additionally, dehydration can lead to constipation (223). Constipation has been associated with decreased *Bacteroides, Roseburia*, and *Coprococcus*and increased abundances of genes involved in gas production (224). Furthermore, stool consistency and transit time are linked to the diversity and composition of the gut microbiota (225). Dehydration also increases gastrointestinal distress symptoms (218), suggesting that insufficient fluid replacement affects gut function and may impact the gut microbiota.

The gut microbiota may also influence hydration status via cellular transport of solutes through the gastrointestinal mucosa (22). Hydration status biomarkers, including copeptin, urine volume, and urine nitrogen concentration, are associated with substrate utilization and energy expenditure (226) as well as long-term health outcomes such as metabolic syndrome, diabetes, obesity, kidney disease, and heart disease (227) and may therefore be useful measurements to assess the relation of the gut microbiota with hydration and health outcomes. These associations would help assess the effects of the gut microbiota on hydration status, or vice versa, and subsequent effects on substrate utilization and gastrointestinal distress in athletes during competition, both of which could potentially impact performance.

Additionally, carbohydrate, electrolyte, and energy beverages are commonly used by endurance athletes (228) but, to our knowledge, no studies have investigated the effects of carbohydrate or concentrated sports drinks on the gut microbiota (17). However, intake of both caloric and low/noncaloric sweeteners and food emulsifiers commonly contained in these beverages may have harmful, proinflammatory effects (229). Both sucralose and emulsifiers such as carrageenan have been shown to trigger proinflammatory responses, including upregulation of TNF-α as well as increased gastrointestinal permeability in both humans and animal models (146, 229). Ultimately, the effects of low/noncaloric sweeteners on the gut microbiota of athletes remain unclear (138, 230–233).

### Sport supplements

To support and enhance athletic performance, athletes frequently consume nutritional supplements that may also have additional, unintended, impacts on the gut microbiota (17). Some of these, such as protein supplements, BCAAs, taurine, ɷ-3 fatty acids, vitamin D, and probiotics, have already been discussed. However, other commonly used supplements include antioxidants, nitrates, sodium bicarbonate, creatine, B-alanine, and caffeine (17, 234).

While some degree of exercise-induced oxidative stress is necessary for muscle adaptation, excessive ROS concentrations may compromise health, immunity, and recovery (17). Polyphenols are plant-derived compounds commonly used for their antioxidant properties to mitigate excessive oxidative stress in athletes (235). However, the bioavailability, absorption, and effects of polyphenols often depend on their conversion by the gut microbiota into more bioavailable, bioactive metabolites (236, 237). Additionally, polyphenols exert prebiotic-like effects on the gut microbiota composition by increasing the abundances of commensal bacteria, including *Bifidobacterium, Lactobacilli, Akkermansia muciniphila, Faecalibacterium prausnitzii*, and *Roseburia* spp. (237). Therefore, in addition to direct effects on reducing excess ROS, polyphenols may improve recovery and performance via their effects on the gut microbiota and the production of microbial metabolites.

Nitrates, mainly in the form of beetroot juice, improve athletic performance via increased oxygen uptake efficiency by skeletal muscle (238). Conversion of dietary nitrate to nitrite may also influence the gut microbiota composition via antimicrobial properties and modulation of intestinal permeability (238). However, it is difficult to isolate the role of nitrates from other compounds, such as polyphenols provided by vegetable intake (238). Certain bacteria can also utilize nitrate as a nutrient, which may increase bioavailability in skeletal muscle and contribute to its ergogenic effect (238).

Sodium bicarbonate is used to enhance buffering capacity, thus mitigating the increase in intracellular acidosis during intense exercise (239). Bicarbonate-rich mineral water consumption increases *Christenellaceae, Bacteroidaceae*, and *Erysipelotrichaceae* and decreases *Bifidobacteriaceae* (240). While higher abundance of *Christenellaceae* has been reported in lean individuals relative to individuals with obesity (241), it is unclear whether changes in the gut microbiota resulting from sodium bicarbonate supplementation may contribute to its ergogenic effects during exercise.

Creatine increases the muscle phosphocreatine reservoir, enhancing rapid ATP regeneration during high-intensity exercise (234). To our knowledge, there are no studies of the effects of creatine supplementation on the gut microbiota. Higher doses of creatine (≥10g) increase gastrointestinal distress and the risk of diarrhea (242). However, lower doses of creatine do not affect gastrointestinal symptoms (242) and research in mice suggests that glycine amidinotransferase (GATM), the enzyme that catalyzes the rate-limiting step of creatine biosynthesis, has a beneficial effect on gastrointestinal barrier integrity (243).

B-alanine is the rate-limiting precursor for carnosine synthesis, and supplementation is used to elevate muscle carnosine concentration, providing a benefit for high-intensity exercise (234). The effects of B-alanine supplementation on the gut microbiota or the effects of the gut microbiota on B-alanine supplementation efficacy have not been investigated. However, certain bacteria, including *Lactobacilli* and *Streptococcus thermophilus*, have functional genes capable of B-alanine metabolism (244). Furthermore, animal models using antibiotic treatment and stress induced changes in microbial metabolism of B-alanine (245, 246). Therefore, it is plausible that the gut microbiota may influence the ergogenic effects of B-alanine supplementation.

Caffeine is widely used to reduce perceived effort, fatigue, or pain during exercise (234). Caffeine can be consumed in coffee, tea, energy drinks, pills, or foods. While some research has shown modest effects of coffee on the gut microbiota, such as increases in *Bifidobacterium* and *Bacteroides* (247, 248), coffee and tea contain complex mixtures of other compounds, such as polyphenols and chlorogenic acid, that may also impact the gut microbiota. A study in mice investigated the effects of coffee or coffee components (i.e., caffeine or chlorogenic acid) on the gut microbiota and demonstrated that caffeine increased butyrate and propionate (249). However, chlorogenic acid induced greater increases in acetate, propionate, and butyrate, while coffee had no significant effect, although another study in rats revealed an increase in SCFAs in response to coffee intake (250). Therefore, it is difficult to determine the potential for the gut microbiota and SCFAs to mediate the ergogenic effects of caffeine or coffee intake.

Overall, there is evidence to suggest that supplements commonly used by athletes may also affect the gut microbiota and the production of metabolites such as SCFAs. The implications of these changes in the gut microbiota on the ergogenic effects of these supplements are unclear but could involve mediation of effects via the gut–muscle axis.

## Future Directions

There is currently a lack of research in humans on the interaction between the gut microbiota and exercise, particularly in combination with a controlled diet, which is a significant confounding factor. Researchers should implement validated approaches to assess acute (Automated Self-Administered 24-hour [ASA-24] dietary recall) and habitual dietary intake (Food-Frequency Questionnaire [FFQ]), which also allow for the calculation of standardized values such as the Healthy Eating Index (HEI). Dietary quality, commonly measured using the HEI, is associated with better physical performance (251), although it has been proposed that an Athlete Diet Index targeted specifically at assessing dietary quality for athletes may be more relevant (252). In terms of nutrient intake, higher protein intake in athletes compared with sedentary controls has been documented (83). However, an emphasis on protein, carbohydrate, and fat intake differs by sport modality (84, 253), gender (254), and as a result of fluctuations in training (255). Therefore, accurate measurement of both nutrient content and diet quality will help separate the effects of sport on the gut microbiota from other confounding factors. Studies should also record fluid intake or measure hydration biomarkers (e.g., copeptin) to determine whether hydration status affects the gut microbiota or vice versa. Additionally, the effects of diet and exercise on the gut microbiota are often transient and do not persist after completion of the intervention (23, 256, 257). This suggests that long-term lifestyle habits are necessary to induce stable shifts in the gut microbiota. Alternatively, certain interventions or interventions during critical development windows may have more lasting effects on the gut microbiota, although this requires further investigation.

Although animal models are useful because factors such as diet can be stringently controlled and tissue samples are available to study mechanisms, differences in gastrointestinal physiology, microbiota compositions, effects of genetic background in mice, coprophagy, housing conditions, and feeding, as well as insufficient numbers of “donor microbiomes” in the case of human microbiota transplants in rodents all limit the translation of rodent research (258, 259). Future research should focus on using a tiered approach in which human clinical trials are used to identify target bacteria that may benefit athletic performance and animal and in vitro studies are used to determine causality and mechanisms. Human trials may then be used again to determine whether supplementation with the identified bacteria or implementation of dietary practices (e.g., prebiotics/nondigestible carbohydrates, ɷ-3 fatty acid supplements, type/amount of protein intake) that enhance bacterial abundance and/or functionality are beneficial for athletic performance.

Clinical studies investigating the effects of high-protein diets, whole-food protein sources, and protein supplements in the context of a controlled diet are needed to determine the impact of these dietary patterns and components on the gut microbiota in athletes. Additionally, more research is needed to clarify the effects of amounts and types of dietary fat on the gut microbiota and subsequent microbiota-mediated (e.g., via bile acids) effects on exercise performance. This research should consider differences among athletes practicing sports of different durations and intensities and effects in female athletes, as fat oxidation during exercise is higher in women versus men (260). Research is also needed to establish the potential detrimental effects of a diet low in nondigestible carbohydrates on the athletic gut microbiota as well as the potential beneficial effects of different types, doses, and timing of fiber intake and other candidate prebiotics, in whole foods or as isolated supplements, on athlete health and performance while addressing issues of tolerability and gastrointestinal distress. To assess the role of microbial metabolites, future studies should consider the use of intrinsically labeled SCFAs to assess systemic availability and their incorporation into biologically relevant molecules (18). While some research suggests a potential ergogenic effect of probiotic strains in athletes (2, 36, 156, 261), confirmatory trials to replicate findings are rare. Therefore, correlations of single strains or multi-strain formulations with certain performance or health outcomes are primarily based on a single study (172). More evidence is needed to clarify the potential for ingestion of probiotics or fermented foods to enhance athletic performance. Additionally, more research is needed with larger and more diverse sample populations to determine the specific strains or combinations of strains that may induce specific, desired responses in athletes and potential modification of effects by individual factors, such as gender, as this has been reported to impact gastrointestinal structure, function, and microbiota at rest and during exercise (35). Similarly, response to prebiotics, probiotics, and dietary strategies such as a low-FODMAP or gluten-free diet may differ based on an individual's baseline microbiota composition (8, 262), indicating that researchers must take a precision nutrition approach to account for interindividual differences that may influence the efficacy of avoidance of certain dietary components. This may also be true of the microbial and ergogenic response to dietary supplements, although more research is needed to understand the interaction between sports supplements and the gut microbiota.

It is increasingly recognized that the responses of an individual's gut microbiota to diet are personalized depending on characteristics such as the presence or abundance of keystone species (e.g., *Ruminococcus bromii* or *Prevotella copri*) (155, 263) or metabotypes (264). Interindividual variability in microbial responses then contributes to variability in metabolic responses (e.g., glycemic response) and health outcomes (e.g., weight loss) (7, 8). Therefore, dietary strategies require a nuanced approach to optimize health via the gut microbiota. To capture this complexity, future research should also integrate other “omics” data to determine potential metabolites, genes, and epigenetic modifications that may cause, contribute to, mediate, or modulate the effects of diet and exercise on the gut microbiota (265–267). The use of “omics” data coupled with machine-learning methods has the potential to uncover novel associations between the gut microbiota and its metabolites, diet, and athletic performance, as well as predict personalized responses to dietary strategies (16). The impacts of these findings include the potential for enhanced performance in athletes and improved health, particularly gastrointestinal and respiratory health. Additionally, the research will lead to a greater understanding of the interaction between the gut microbiota, diet, and human health that may have implications and applications that extend beyond the athletic population to benefit the health of all.

## Conclusions

To achieve optimum performance, athletes must fuel, train, and utilize their entire supraorganism, including their gut microbiota, by implementing gut-centric dietary strategies (**Text Box 2**). There is a growing body of research on the role of the gut microbiota in sport and performance. Current evidence suggests that the gut microbiota may contribute to the effects of dietary intake on athletic performance via production of metabolites (e.g., SCFAs, secondary bile acids), influence on gastrointestinal physiology (e.g., nutrient absorption, barrier integrity, motility, gas production), and immune modulation (e.g., pathogen inhibition, GALT). Common dietary strategies in athletes, such as high protein and simple carbohydrate intake, low intake of nondigestible carbohydrates, and food avoidance, may adversely impact the gut microbiota and predispose athletes to gastrointestinal distress and thus impair performance. Conversely, intake of adequate dietary fiber, a variety of protein sources, and emphasis on unsaturated fats, especially ɷ-3 fatty acids, as well as supplementation with pre-, pro-, and synbiotics, have shown promising results in optimizing the health of the athlete and their gut microbiota with potential beneficial effects on performance.

Text Box 2. Summary

• Diet and exercise affect the composition and function of the gut microbiome via substrate availability and physiological changes to the gastrointestinal environment.

• Sport-centric dietary strategies such as high protein, carbohydrate loading, and FODMAP restriction as well as gut-centric dietary strategies such as pro-, pre-, and synbiotics all represent opportunities to impact both the gut microbiota and athletic performance.

• High-protein diets and use of protein supplements show a greater effect on microbial metabolites than on the gut microbiota composition. The gut microbiota may contribute to muscle protein anabolism and function by modulating protein absorption and utilization.

• High-fat and saturated fat intake are associated with a proinflammatory gut microbiota composition, although ɷ-3 fatty acids promote SCFA production. Effects of these changes on athletic performance is inconclusive.

• Intake of highly digestible carbohydrates at the expense of fiber has detrimental effects on the gut microbiota, whereas SCFAs produced by the gut microbiota from dietary fiber are positively associated with muscle function.

• Pro-, pre-, and synbiotics can alter the gut microbiota and positively affect athletic performance and recovery. Variability in strains, doses, and other individual factors makes it difficult to identify the ergogenic effects of these gut-centric dietary strategies.

• The gut microbiota influences the absorption of certain micronutrients, including calcium, that are important for aspects of athlete health and performance, such as bone health.

• Short-term or pre-exercise avoidance of certain foods or food groups, such as FODMAPs or gluten, may be warranted for some individuals but the long-term effects of these strategies on the athletic gut microbiota and performance are unclear.

• Energy deficiency or excess both influence the gut microbiota. The gut microbiota and microbiota-based therapies may help alleviate detrimental effects of both extremes including gastrointestinal symptoms and compromised bone density.

• There is limited evidence on the effect of hydration status or sports drinks on the gut microbiota, although dehydration is associated with constipation and gastrointestinal symptoms that affect or indicate effects on the gut microbiota.

• Sports supplements are used for their ergogenic effects but their effects on the gut microbiota are unclear and warrant further research.

## Notes

This work was partially supported by Danone Research.

Author disclosures: The authors report no conflicts of interest.

Abbreviations used: BCAA, branched-chain amino acid; FODMAP, fermentable oligo-, di-, monosaccharides and polyols; FXR, farnesoid X receptor; GALT, gut-associated lymphoid tissue; HEI, Healthy Eating Index; IGF-I, insulin-like growth factor I; IMTG, intramuscular triglyceride; IPE, inulin-propionate ester; PAG, phenylacetylglycine; ROS, reactive oxygen species; TGR5, G protein–coupled receptor 5; TMAO, trimethylamine-N-oxide; $\dot{V}{{\rm{O}}_2}\max $, maximal oxygen uptake.
